# Annual Report of the Registrar-General

**Published:** 1857-01

**Authors:** 


### ANNUAL REPORT OF THE REGISTRAR-GENERAL.

The Registrar-General has brought out his Seventeenth Annual Report, containing a letter from Dr. Farr, which is, as usual, full of valuable and interesting matter. In 1854, not fewer than 113,576 persons died of zymotic diseases. The fatality stands in the following order: deaths in 10,000 living; cholera, 11; diarrhœa, 11; scarlatina, 10; typhus, 10; hooping-cough, 5; measles 5; croup, 2; small-pox, 1·5; dysentery, 1; erysipelas, 1. The letter goes to substantiate the view as to the propagation of cholera by impure water.

